# Syndecans, Exostosins and Sulfotransferases as Potential Synovial Inflammation Moderators in Patients with Hip Osteoarthritis

**DOI:** 10.3390/ijms25084557

**Published:** 2024-04-22

**Authors:** Matko Rošin, Nela Kelam, Ivana Jurić, Anita Racetin, Marin Ogorevc, Brieuc Corre, Davor Čarić, Natalija Filipović, Katarina Vukojević

**Affiliations:** 1 Surgery Department, Orthopaedics and Traumatology Division, University Hospital of Split, Spinciceva 1, 21000 Split, Croatia; mrosin@kbsplit.hr (M.R.); dcaric@kbsplit.hr (D.Č.); 2 Department of Anatomy, Histology and Embryology, University of Split School of Medicine, Soltanska 2, 21000 Split, Croatia; nela.kelam@mefst.hr (N.K.); amuic@mefst.hr (A.R.); marin.ogorevc2@gmail.com (M.O.); natalija.filipovic@mefst.hr (N.F.); 3 Department of Emergency Medicine, University Hospital of Split, Spinciceva 1, 21000 Split, Croatia; ivanajuric55555@gmail.com; 4 Faculty of Medicine and Health Sciences, University of Brest, 29200 Brest, France; brieuc.c123@gmail.com; 5 Center for Translational Research in Biomedicine, University of Split School of Medicine, Soltanska 2, 21000 Split, Croatia

**Keywords:** osteoarthritis, synovial membrane, syndecan, exostosin, sulfotransferase

## Abstract

The gradual deterioration of articular cartilage was thought to be the central event in osteoarthritis (OA), but recent studies demonstrated the importance of low-grade synovitis in the progression of OA. The Syndecan (SDC) family of membrane proteoglycans is known to be involved in the regulation of inflammation, but there is limited evidence considering the role of syndecans in OA synovitis. Our study aimed to investigate the hip OA synovial membrane expression patterns of SDC1, SDC2 and SDC4, as well as exostosins and sulfotransferases (enzymes involved in the polymerisation and modification of syndecans’ heparan sulphate chains). Synovial membrane samples of patients with OA (24) were divided into two groups according to their Krenn synovitis score severity. The immunohistochemical expressions of SDC1, SDC2, SDC4, EXT1, EXT2, NDST1 and NDST2 in synovial intima and subintima were then analysed and compared with the control group (patients with femoral neck fracture). According to our study, the immunoexpression of SDC1, NDST1 and EXT2 is significantly increased in the intimal cells of OA synovial membrane in patients with lower histological synovitis scores and SDC4 in patients with higher synovitis scores, in comparison with non-OA controls. The difference in the expression of SDC2 among the OA and non-OA groups was insignificant. SDC1, SDC4, NDST1 and EXT2 seem to be involved as inflammation moderators in low-grade OA synovitis and, therefore, should be further investigated as potential markers of disease progression and therapeutic goals.

## 1. Introduction

Osteoarthritis (OA) is the most common form of arthritis. It can affect any joint but typically involves the knees and hips. It is one of the most common and important causes of chronic pain and disability. It severely impacts patients’ quality of life and increases the socioeconomic burden [1,2,3]. Globally, hip and knee OA was ranked among the highest contributor to global disability in disability-adjusted life years [4,5,6].

According to the Osteoarthritis Research Society International’s definition of OA, OA is a disorder of mobile joints illustrated by the cellular degradation of the extracellular matrix through micro- and macro-injuries that initiate adaptive repair mechanisms, including pro-inflammatory pathways of innate immunity. The disorder manifests itself first at the molecular level (impaired metabolism of joint tissues), and then through anatomical and physiological impairment (cartilage degradation, bone remodelling, osteophyte formation, and joint inflammation), resulting in loss of joint function and disease manifestation [5]. It is important to note that OA is not a pathophysiologically exclusive disease but rather a diverse syndrome with altered clinical phenotypes that affects all joint structures, eventually leading to common clinical manifestations [6,7,8]. OA can be divided into primary (idiopathic) and secondary OA [9]. Secondary OA is commonly caused by post-traumatic, dysplastic, infectious, inflammatory, or biochemical events [10].

Primary OA was long thought to be a disease caused by prolonged and overloaded articular cartilage wear and tear. However, the current understanding of the disease shows that pathological changes involve cartilage, bone, synovium, ligaments, fat tissues, menisci, and neurological pathways involved in pain processing. The changes occur not only due to mechanical overloads, but also due to metabolic and genetic factors that result in inflammation [11]. Therefore, synovial inflammation is often present in both early OA and advanced OA and is implicated in the OA development and progression. Synovial cells produce molecules that enable synovial inflammation and lead to cartilage damage during OA progression [12]. The histological changes observed in the OA synovium donors usually include heterogeneous inflammatory landscapes with a patchy distribution of the proliferation of the synovial lining and the immune cell infiltration [13]. Compared with rheumatoid arthritis patients (RA), the grade of inflammatory cell infiltration in the synovium is significantly lower in patients with OA [14]. However, the mechanism that triggers synovitis remains unclear. Accordingly, OA synovitis might be initiated by chondrocyte cartilage degradation products and mediators [15].

The inflammation of the synovial tissue that is observed in OA results in detectable synovitis determined through imaging, arthroscopy, or histological analysis. Despite the inflammation of the synovium, OA is mostly classified as a disorder without inflammation because in OA synovial fluid, the leukocyte count is typically below the threshold for an “inflammatory” disorder [15]. Numerous studies have associated OA with various inflammatory mediators like cytokines, growth factors, chemokines, adipokines, neuropeptides, prostaglandins, leukotrienes, nitric oxide, and several therapeutic agents that target inflammatory components have shown some progress in animal models. Positive effects of sulphated biofermentative chondroitin (BC) were observed in in vitro models resembling OA pathology [16]. However, their potential in human OA remains to be validated [17].

Syndecans (SDCs) are a family of four similar transmembrane heparan-sulphate proteoglycans, and due to their mutual similarity, syndecan-1 and -3 are members of one subgroup, and syndecan-2 and -4 are members of another [18,19]. Syndecans are capable of interacting with various extracellular ligands [20], thus initiating various biological signals related to cell adhesion, angiogenesis [21], inflammation and tissue repair [22]. Syndecans in inflammation regulate leukocyte extravasation and cytokine function. They also participate in the controlled progression of inflammation [23]. In addition to the transmembrane form, syndecans also exist as soluble extracellular domains [24]. The proteolytic juxtamembranous cleavage of syndecans is associated with inflammation, mostly due to the enzymatic action of sheddases, e.g., matrix metalloproteinases (MMPs) or a disintegrin and metalloproteinase with thrombospondin motifs (ADAMTS) [25]. Syndecans released into the extracellular matrix can reduce signal transduction in the cells, saturate ligands and trigger additional signalling pathways as circulating effectors [22]. Although syndecan-4 is hardly expressed in articular cartilage, it is strongly upregulated in human OA and in animal OA degenerative joint damage models [20,26,27]. Potentially, a good predictor for OA severity can be MMP-9-mediated syndecan-4 shedding, which is increased in the synovial fluid of OA patients but not in the serum [28]. The significance of the expressions of other syndecans in OA cartilage has not been established in existing studies [20]. Syndecan-1 expression was noted in the mononuclear infiltrates of synovial tissue from RA patients and patients with psoriatic arthritis [29], but there are no studies that analyse the expressions of syndecan-1 and syndecan-4 in the synovium of patients with hip OA. The exostosin family of glycosyltransferases mediates the synthesis of heparan sulphate proteoglycans (to which syndecans also belong)—it has been recognised that both exostosin-1 (EXT1) and exostosin-2 (EXT2) are needed in vivo for heparan sulphate chain elongation [30]. EXT1 and EXT2 mutations are associated with the development of hereditary multiple osteochondroma [31]. N-deacetylase/N-sulfotransferase (NDST) catalyses the modification of heparan sulphate oligosaccharides. Pro-inflammatory cytokines can increase the expression of NDST, ultimately leading to the extravasation of leukocytes at the site of inflammation [32]. To our knowledge, there is no evidence regarding the expressions of exostosins and NDSTs in the synovial membrane of OA patients. Therefore, our study aimed to explore the immunoexpression of syndecans, exostosins and NDSTs in the hip synovium of OA patients, relate these to the standard histological synovitis grading score (according to Krenn [33]) and compare it to that of healthy controls (patients with femoral neck fracture).

## 2. Results

**Figure 1:**
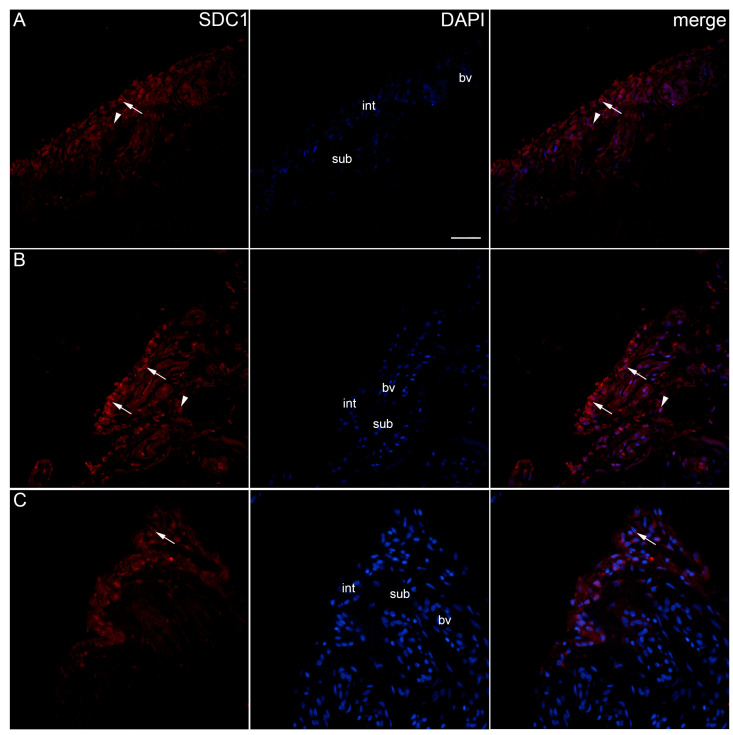
Immunoexpression of SDC1 (syndecan-1) in the synovial membrane of patients with hip osteoarthritis (OA). (**A**) Hip synovium of patients without OA (controls), (**B**) hip synovium of patients with low synovitis score (Krenn score 0–2), (**C**) hip synovium of patients with higher synovitis score of OA (Krenn score ≥ 3); int—intima, sub—subintima, bv—blood vessel. SDC1-positive cells (red signal) can be seen in the intima (arrows) and subintima (arrowheads) of all analysed groups (**A**–**C**). 4′,6-diamidino-2-phenylindole (DAPI) stains all cell nuclei blue. SDC1 merged with DAPI nuclear staining is displayed in the far right column (merge). Images were taken at a magnification of ×40. The scale bar is 100 μm and refers to all images.

**Figure 2:**
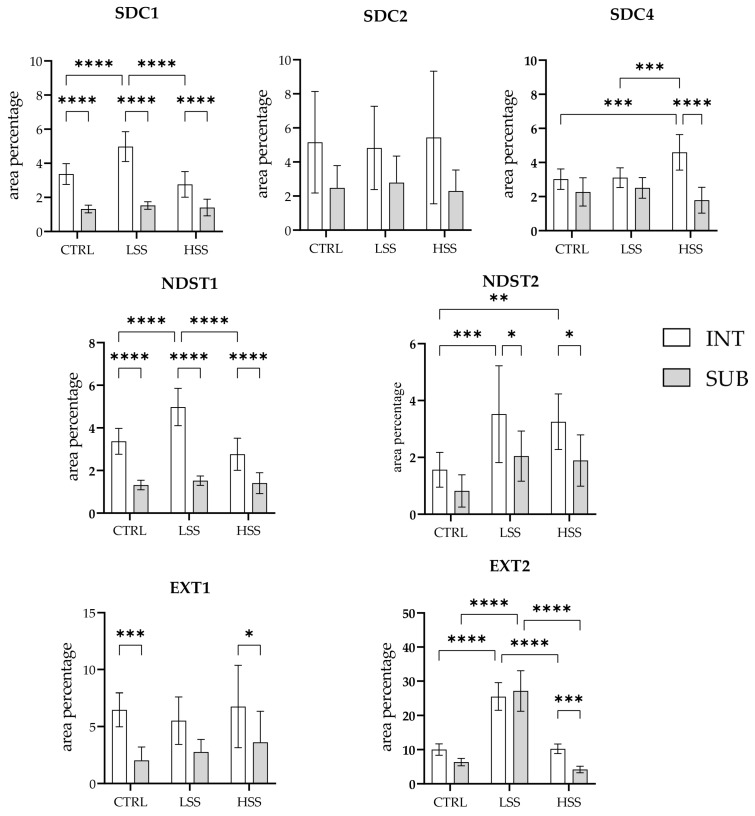
Statistical analyses of protein immunoexpression in the synovial membrane of patients with hip osteoarthritis (OA). INT—intima, SUB—subintima, CTRL—controls, LSS—low synovitis score of OA (Krenn score 0–2), HSS—higher synovitis score of OA (Krenn score ≥ 3). All analyses were performed using two-way ANOVA with Tukey’s post hoc test. The bars of the graphs represent the mean area percentage of the immunofluorescence signal of the analysed proteins, while the error bars represent the standard deviation. Asterisks mark significant differences: * *p* < 0.05, ** *p* < 0.01, *** *p* < 0.001, **** *p* < 0.0001.

After standard histological staining, we divided OA participants into two groups, based on histopathological synovitis intensity according to Krenn: in the OA group with a low synovitis score 0–2 (LSS OA), we had 10 participants (average score: 1.33), and in the OA group with a higher synovitis score ≥ 3 (HSS OA), we had 14 participants (average score: 3.46). All OA participants had an average synovitis score of 2.59 (range 0–5), which is higher than described in the original Krenn scoring profile for OA but with a similar distribution among groups [33]. In the control group, we had 10 participants with an average synovitis score of 0.62 (range 0–1). When analysing the expression pattern of SDC1 in the synovial membranes of patients with hip OA and healthy controls, positive cells were found in the intima and subintima of all the analysed groups (Figure 1). There was no significant reactivity in the subintimal blood vessels. Statistically, the intima displayed higher immunoexpression of SDC1 (*p* < 0.0001) than the subintima of all analysed groups. The intima of the low-synovitis-score group had greater positivity (*p* < 0.0001) compared to both the control and higher-synovitis-score groups (Figure 2).

**Figure 3:**
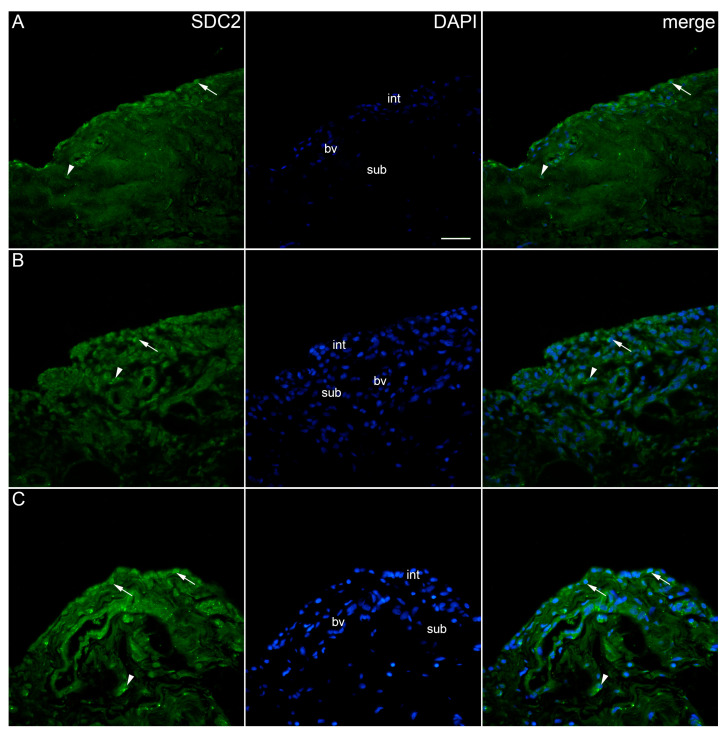
Immunoexpression of SDC2 (syndecan-2) in the synovial membrane of patients with hip osteoarthritis (OA). (**A**) Hip synovium of patients without OA (controls), (**B**) hip synovium of patients with low synovitis score (Krenn score 0–2), (**C**) hip synovium of patients with higher synovitis score of OA (Krenn score ≥ 3); int—intima, sub—subintima, bv—blood vessel. SDC2-positive cells (green signal) can be seen in the intima (arrows) and subintima (arrowheads) of all analysed groups (**A**–**C**). SDC2 positivity can be seen in some cells of subintimal blood vessels (**A**–**C**). 4′,6-diamidino-2-phenylindole (DAPI) stains all cell nuclei blue. SDC2 merged with DAPI nuclear staining is displayed in the far right column (merge). Images were taken at a magnification of ×40. The scale bar is 100 μm and refers to all images.

SDC2 demonstrated positivity in the intima and subintima of the synovial membrane of patients with hip OA and healthy controls (Figure 3). SDC2 positivity could occasionally be seen in some subintimal blood vessel cells. There were no significant differences in SDC2 expression between the analysed groups (Figure 2).

**Figure 4:**
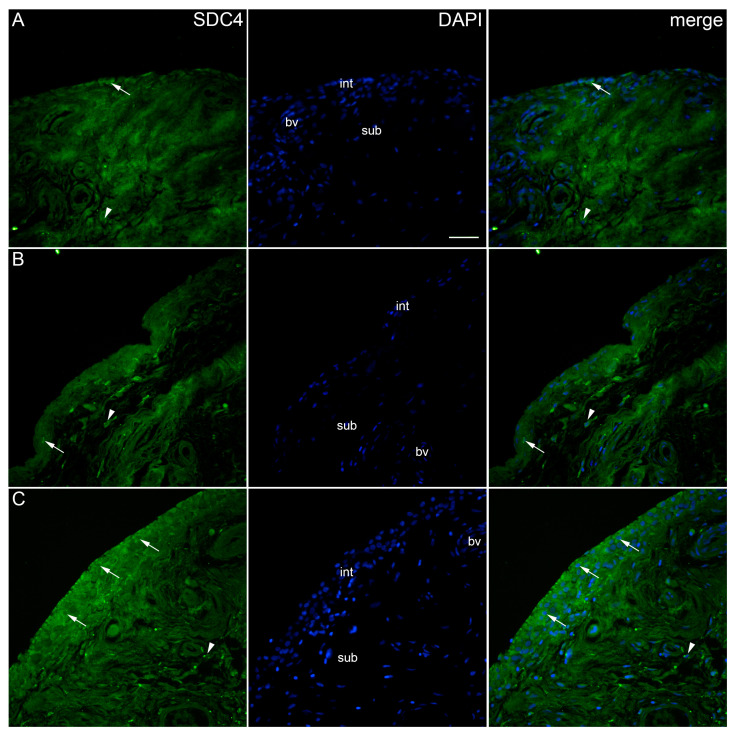
Immunoexpression of SDC4 (syndecan-4) in the synovial membrane of patients with hip osteoarthritis (OA). (**A**) Hip synovium of patients without OA (controls), (**B**) Hip synovium of patients with low synovitis score of OA (Krenn score 0–2), (**C**) hip synovium of patients with higher synovitis score of OA (Krenn score ≥ 3); int—intima, sub—subintima, bv—blood vessel. SDC4-positive cells (green signal) can be seen in the intima (arrows) and subintima (arrowheads) of all analysed groups (**A**–**C**). 4′,6-diamidino-2-phenylindole (DAPI) stains all cell nuclei blue. SDC4 merged with DAPI nuclear staining is displayed in the far right column (merge). Images were taken at a magnification of ×40. The scale bar is 100 μm and refers to all images.

When analysing the expression of SDC4 in the synovial membrane of patients with hip OA and healthy controls, we observed positive cells in the intima and subintima of all analysed groups (Figure 4). There was no significant SDC4 immunoreactivity in the blood vessels. The intima of the higher-synovitis-score group showed a significantly higher expression compared to both the subintima and intima of the other analysed groups (*p* < 0.0001) (Figure 2).

**Figure 5:**
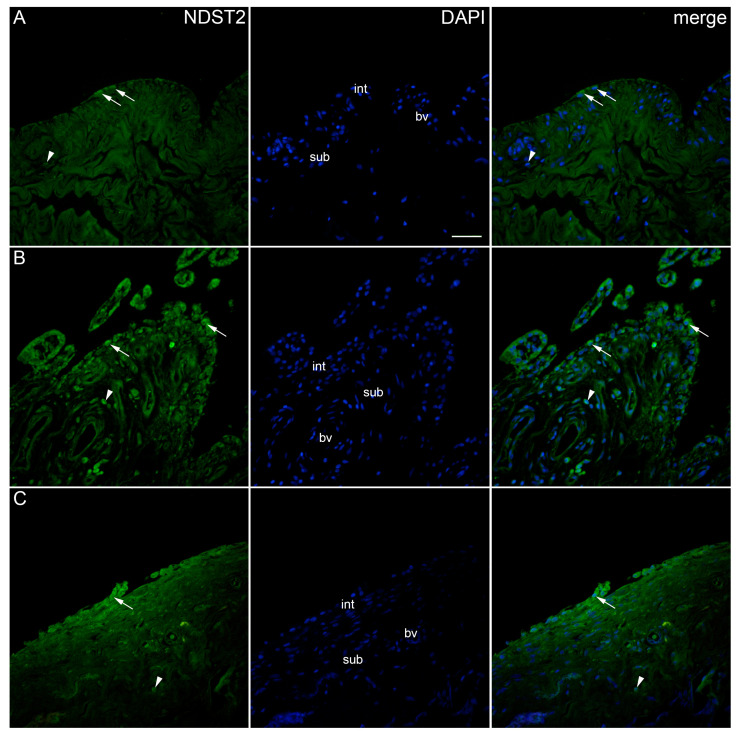
Immunoexpression of NDST1 (N-deacetylase/N-sulfotransferase 1) in the synovial membrane of patients with hip osteoarthritis (OA). (**A**) Hip synovium of patients without OA (controls), (**B**) hip synovium of patients with low synovitis score of OA (Krenn score 0–2), (**C**) hip synovium of patients with higher synovitis score of OA (Krenn score ≥ 3); int—intima, sub—subintima, bv—blood vessel. NDST1-positive cells (green signal) can be seen in the intima (arrows) and subintima (arrowheads) of all analysed groups (**A**–**C**). Strong positivity can be observed in the subintimal blood vessels of the mild OA group (**B**). 4′,6-diamidino-2-phenylindole (DAPI) stains all cell nuclei blue. NDST1 merged with DAPI nuclear staining is displayed in the far right column (merge). Images were taken at a magnification of ×40. The scale bar is 100 μm and refers to all images.

The immunoexpression of NDST1 was observed in the synovial membrane of patients with hip OA (Figure 5). NDST1-positive cells were seen in the intima and subintima of all analysed groups. Strong positivity was observed in the subintimal blood vessels of the mild synovitis group. Statistically, the intima displayed higher immunoexpression (*p* < 0.0001) than the subintima of all analysed groups. The intima of the mild-synovitis-score group had greater positivity (*p* < 0.0001) compared to both the control and higher-synovitis-score groups (Figure 2).

**Figure 6:**
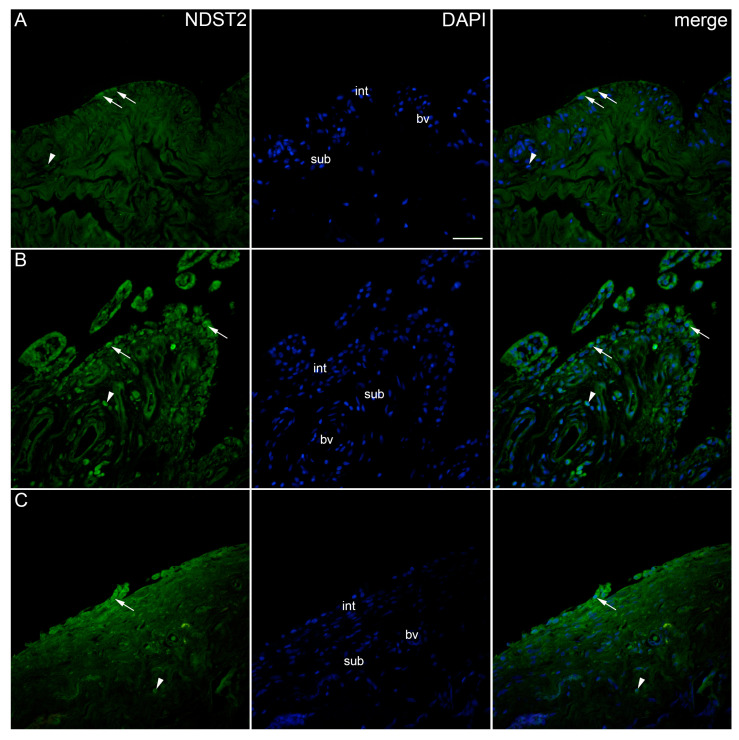
Immunoexpression of NDST2 (N-deacetylase/N-sulfotransferase 2) in the synovial membrane of patients with hip osteoarthritis (OA). (**A**) Hip synovium of patients without OA (controls), (**B**) hip synovium of patients with low synovitis score of OA (Krenn score 0–2), (**C**) hip synovium of patients with higher synovitis score of OA (Krenn score ≥ 3); int—intima, sub—subintima, bv—blood vessel. NDST2-positive cells (green signal) can be seen in the intima (arrows) and subintima (arrowheads) of all analysed groups (**A**–**C**). 4′,6-diamidino-2-phenylindole (DAPI) stains all cell nuclei blue. NDST2 merged with DAPI nuclear staining is displayed in the far right column (merge). Images were taken at a magnification of ×40. The scale bar is 100 μm and refers to all images.

NDST2 showed positive expression in the synovial membrane of patients with hip OA and controls (Figure 6). NDST2-positive cells were found in the intima and subintima of all analysed groups. However, compared to NDST1, there was no significant reactivity in the blood vessels. Statistically, the expression of NDST2 was significantly lower in the synovial membranes of the controls compared to both the low-synovitis-score and higher-synovitis-score groups (*p* = 0.001, *p* = 0.006, respectively). The intima of both OA groups demonstrated significantly higher positivity regarding the subintima (*p* = 0.022 and *p* = 0.043, respectively) (Figure 2).

**Figure 7:**
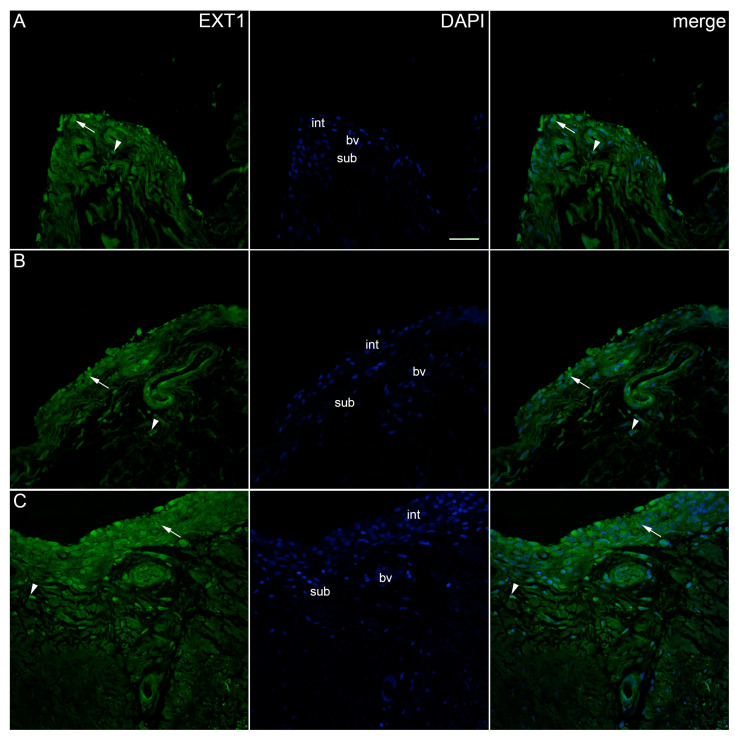
Immunoexpression of EXT1 (exostosin 1) in the synovial membrane of patients with hip osteoarthritis (OA). (**A**) Hip synovium of patients without OA (controls), (**B**) hip synovium of patients with low synovitis score of OA (Krenn score 0–2), (**C**) hip synovium of patients with higher synovitis score of OA (Krenn score ≥ 3); int—intima, sub—subintima, bv—blood vessel. EXT1-positive cells (green signal) can be seen in the intima (arrows) and subintima (arrowheads) of all analysed groups (**A**–**C**). Endothelial cells of subintimal blood vessels demonstrate strong EXT1 positivity, especially in OA patients (**B**,**C**). 4′,6-diamidino-2-phenylindole (DAPI) stains all cell nuclei blue. EXT1 merged with DAPI nuclear staining is displayed in the far right column (merge). Images were taken at a magnification of ×40. The scale bar is 100 μm and refers to all images.

The expression pattern of EXT1 in the synovial membrane of patients with hip OA were analysed and compared to that of healthy controls (Figure 7). EXT1-positive cells were seen in the intima and subintima of all analysed groups. The endothelial cells of the subintimal blood vessels demonstrated strong EXT1 positivity, especially in OA patients. There were no statistically significant differences in EXT1 expression between the analysed groups. However, the intima of the control and higher-synovitis-score OA group displayed higher positivity than the subintima (*p* = 0.001, *p* = 0.029, respectively) (Figure 2).

**Figure 8:**
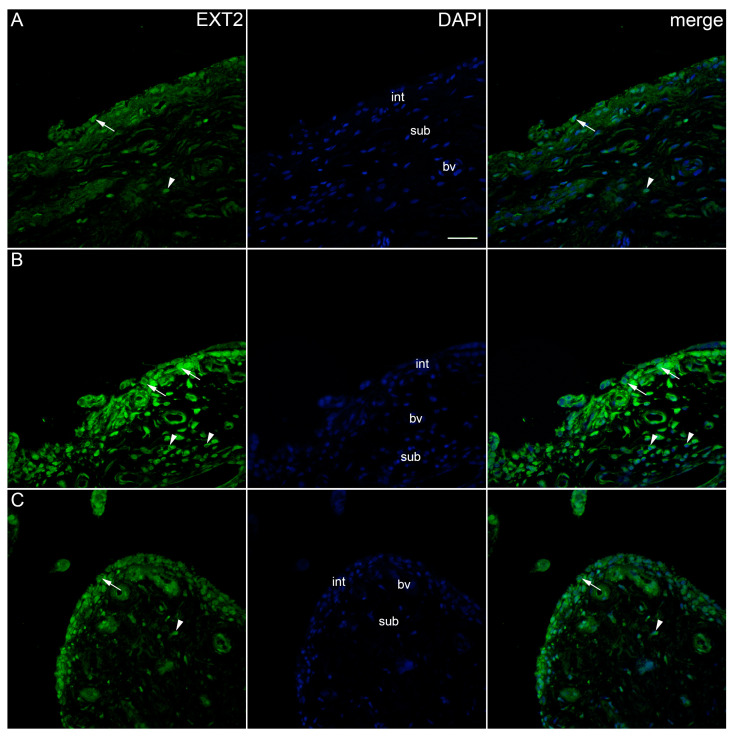
Immunoexpression of EXT2 (exostosin 2) in the synovial membrane of patients with hip osteoarthritis (OA). (**A**) Hip synovium of patients without OA (controls), (**B**) hip synovium of patients with low synovitis score of OA (Krenn score 0–2), (**C**) hip synovium of patients with higher synovitis score of OA (Krenn score ≥ 3); int—intima, sub—subintima, bv—blood vessel. EXT2-positive cells (green signal) can be seen in the intima (arrows) and subintima (arrowheads) of all analysed groups (**A**–**C**). Strong positivity can be observed in the subintimal blood vessels of the mild-OA group (**B**). 4′,6-diamidino-2-phenylindole (DAPI) stains all cell nuclei blue. EXT2 merged with DAPI nuclear staining is displayed in the far right column (merge). Images were taken at a magnification of ×40. The scale bar is 100 μm and refers to all images.

The immunoexpression of EXT2 was found in the synovial membrane of patients with hip OA and healthy controls (Figure 8). The intima and subintima of all analysed groups contained EXT2-positive cells. Strong positivity was observed in the subintimal blood vessels of the higher-synovitis-score group. A statistical analysis showed the significantly higher immunoexpression of EXT2 in both the intima and subintima of the low-synovitis-score group (*p* < 0.0001) compared to that of the other groups. In the case of the higher-synovitis-score group, the intima displayed significantly higher positivity than the subintima (*p* = 0.001) (Figure 2).

## 3. Discussion

Osteoarthritis, as one of the important reasons for chronic pain and disability, carries a large socioeconomic burden through its severe impact on patients’ quality of life [1,34,35]. Synovial inflammation can be present in both early- and advanced-stage OA and is important in the development and progression of OA [36,37,38]. Namely, the intima is made up of epithelial like cells, synoviocytes, and the subintima is made up of connective stromal cells with more or less inflammatory infiltrate, especially around blood vessels in OA. Therefore, each of these regions of synovial membranes (intima vs. subintima) might have the specific significances related to each protein tested in our study. Additionally, synovial cells have been proven to be the main type of cells that initiate and coordinate inflammation and contribute to cartilage damage during OA progression by producing different signal molecules [12]. It has been widely proven that, in addition to a clinical factor such as ageing, obesity, trauma and long mechanical loading, different chemical stimuli are involved in the synovial cell production of different inflammatory mediators associated with tissue-damaging molecular patterns [39]. While it was once considered a consequence of cartilage destruction, today, the low-grade inflammation (LGI) of the synovial membrane is recognised as playing a central role in OA pathophysiology; it is obvious that it contributes to symptom and disease progression and that immunological mechanisms are crucial in driving inflammation and tissue destruction [40,41,42,43]. It is also important to notice that there is growing evidence that synovitis contributes to the generation and maintenance of pain in OA [44,45,46], although the actual mechanism of this connection is still unclear. In order to improve the therapeutic approach and decrease OA-caused damage and pain, there is a need to increase our knowledge and understanding of the role of synovitis in OA onset and progression. Hence, there is consensus about the need for additional studies to reveal chemical signalling pathways between the synovial and inflammatory and reparative cells to improve anti-inflammatory therapies, especially in the early stages of the disease [47]. Concerning all the above-mentioned observations, we aimed to explore the expression and potential role of a group of functionally related inflammatory mediating molecules, including members of the syndecan family (SDC1, SDC2, SDC4), exostosins (EXT1, EXT2) and sulfotransferases (NDST1, NDST2) [19,23,48,49], in the hip synovium of patients with OA, and compare their expressions with those of a control group of patients without OA. Considering the explained importance of low-grade synovitis in the progression of OA, we decided to divide subjects with OA into two subgroups, not based on radiological findings but on the histological universally accepted synovitis score developed by Krenn. In his original series of 212 OA cases, the median value was 2 (range 0–6) [33]. Our study had 24 OA cases with an average Krenn score of 2.69 (range 0–5). We did not use Krenn’s original division into three groups (0–1: no synovitis, 2–4: low-grade synovitis, 5–9: high-grade synovitis) due to the fact that this grading system was primarily devised to distinguish between “inflammatory” (e.g., rheumatoid) and “non-inflammatory” synovitis. In an attempt to increase the quality of the Krenn synovitis scoring system, Najm et al. added immunochemistry biomarkers, thus creating a new score—IMSYC [50]. Interestingly, in their series, a few OA patients also exhibited a higher IMSYC than expected, similar to one noted in the rheumatoid arthritis series, suggesting that a more pronounced inflammatory pattern in OA is occasionally present [51]. Further studies demonstrated that histopathological scores (the Krenn synovitis score and IMSYC), in general, reflect clinical disease activity in patients with advanced-stage rheumatoid arthritis [33], but, as far as we know, there is no evidence of using these scores to quantify OA progression.

When planning this research, we hypothesised that we would find a significant increase in the expressions of all investigated molecules compared to those of the controls and that the increase would be higher in the higher-synovitis-score group than in the lower-synovitis-score group. The results of our study only partially confirmed our hypothesis. The expression of SDC2 and, similarly, the expression of EXT1 did not show a significant difference between the OA group and the control group, and therefore, we can speculate that SDC2 and EXT2 are not mediators of synovitis in OA. A study by Zhu et al. [52] investigating syndecans contributing to acute sepsis-associated lung injury came to similar results: the SDC4 gene expression was significantly higher in the inflammatory group than in the control group, while in both groups, SDC2 levels were similar. Still, it is interesting that in our study, only SDC2 positivity could occasionally be observed in subintimal blood vessels.

SDC1, NDST1 and EXT2 did show significantly higher expressions in OA compared to the non-OA controls, but primarily in the OA group with a low synovitis score. The increases in the SDC1, NDST1 and EXT2 expressions were similar across all groups, and therefore, we can speculate that the rise in SDC1 expression is mediated by NDST1 and EXT2. SDC4, on the other hand, showed a significantly increased expression, primarily in the intima of synovitis with a higher inflammatory score.

Previous studies demonstrated a complex interplay between exostosins and NDST1, where the three enzymes (EXT1, EXT2 and NDST1) might determine NDST activity and impact the final heparan sulphate structure. The overexpression of EXT2 enhanced the NDST1 expression and caused elevated HS sulfation, while EXT1 overexpression had the opposite consequences [48]. Our results are align with these findings because we found that NDST1 and EXT2 have similar expression patterns. In trying to explain the more pronounced expression of SDC1 in lower-grade synovitis than in higher-grade synovitis, we should consider that, similar to cytokines, syndecans could serve as both pro-inflammatory and anti-inflammatory molecules. Experimental data support the anti-inflammatory role of SDC1 [23,53]. SDC1 might attenuate non-infectious inflammatory diseases by inhibiting leukocyte bonds on the activated endothelium. Namely, SDC1 reduces the expression and inhibits the activity of pro-inflammatory factors and can constrain leukocyte infiltration to specific injury sites, such as by removing segregated chemokines, thus facilitating inflammation resolve [54]. To make this interpretation even more complicated, although syndecans might control inflammatory cytokines, the contrary is also possible, with cytokines that can control the expression of syndecans. In some cases, the same cytokines even have opposite effects on the expressions of different syndecans [55]. Therefore, it is reasonable to assume that a feedback mechanism is involved in the expression of syndecans during inflammation [23]. The final confounding fact is that syndecans may also influence each other’s expression with compensatory or opposing effects [23]. Duplancic et al. investigated the expressions of SDCs, EXTs and NDSTs in periodontitis, and both SDC1 and SDC2 were positively aligned with the inflammatory infiltrate, but without correlation for SDC4. The heparan sulphate biosynthesis enzymes (EXT1, EXT2, NDST1, and NDST2) displayed comparable correlation with the gingival tissue inflammatory infiltrate in the periodontitis group (EXT1, EXT2 and NDST1 correlated positively, and NDST correlated negatively) [56]. A study by Chanalaris et al. analysed expression changes in heparan sulphate-related genes in human OA cartilage. Among the syndecan family, SDC2 was, similar to our research, found to not be differentially controlled between the groups. Additionally, the expression of SDC4 was reduced in OA, and the expression of SDC1 was increased (SDC1 was the most strongly upregulated). In the same study, the expressions of EXT1 and EXT2, as well as that of NDST1, were increased in OA cartilage [57]. Hattori et al. investigated the influence of the SDC4 intra-articular knee joint injection of an OA mouse model, and their results could not support the pro-inflammatory effects of exogenous SDC4—low-grade synovitis occurred at 2 and 4 weeks and started to be improved at 6 weeks. However, at any time point, there were no significant differences in the average synovitis scores between the SDC4 and control groups. The same study’s results suggest that the treatment of OA articular cartilage with SDC4 inhibits cartilage degeneration, and the proposed mechanism decreases ADAMTS-5 expression and increases TIMP-3 expression [58]. A study by Echtermeyer et al. found that SDC4 knock-out mice had less severe OA-like cartilage destruction in an experimental model of OA, probably as a result of a decrease in ADAMTS-5 activity [26], but that study did not evaluate the absence of SDC4 on OA synovitis changes. The increase in SDC4 in our study in a group with a higher synovitis score is in line with the results of Zhao et al., who found that SDC4 expression in synovial tissue is similar between rheumatoid arthritis (which typically has a high synovitis score) and OA patients [59].

Finally, the relative expressions of all investigated mediators are more pronounced in the intima than in the subintima of the synovial membrane. This finding is in agreement with the published results of an extensive study that used a single-cell RNA sequencing method conducted by Chou et al. [60] in that TNF, IL1B, IL1A and IL6 as major OA-related cytokines and IGF1 as a major OA-related growth factor were mainly expressed by synoviocytes.

We are aware of some significant limitations of our study. The control group included patients with fractures, thus being, although without clinical signs of OA, not completely healthy individuals due to the fact that they very recently underwent significant hip trauma. Anatomical and patient safety-related issues make sampling the synovial membrane in healthy young individuals or early stages OA almost impossible. A relatively small sample size of the investigated synovial membrane may also play a role because it is proven that low-grade OA synovitis has an occasional distribution according to different amorphological sites of the synovium [61]. We did not investigate the role of soluble (shedded) syndecans in correlation with their synovial cell expression, which might be an important in vivo mechanism of syndecan action [62]. Another limitation of this study is the lack of quantitative methods like Western blot or qRT-PCR used to confirm our immunofluorescence results. Additionally, femur fracture patients for the control are not the optimal choice since this sample might have some mild osteoarthritic changes. Also, it was very difficult to obtain samples from low-grade synovitis in OA. However, this is a usual limitation when human patient samples are involved.

## 4. Materials and Methods

### 4.1. Study Population

Our study was conducted with the permission of the Ethics Committee of the University Hospital Split in Split, Croatia, in accordance with the principles of the Declaration of Helsinki. The clinical part of the research (the recruitment of patients, surgical procedures) was conducted at the Department of Orthopedics and Traumatology of the University Hospital in Split. The laboratory part of the research (the processing and analysis of samples) was carried out at the Department of Anatomy, Histology and Embryology of the University of Split School of Medicine. All patients included in the study agreed to participate and signed a consent document.

All subjects included in the study underwent hip arthroplasty surgery. In the investigated population of patients with osteoarthritis of the hip (HOA), the indication for surgery was based on the usual clinical (unsuccessful conservative treatment with persistence of pain and functional limitations) and radiological criteria for total hip replacement. Exclusion criteria for HOA groups were hip dysplasia, history of hip fracture or infection and history of rheumatic conditions. The control group consisted of patients with an indication for hip arthroplasty due to a recent femoral neck fracture, provided that they had no radiological signs of osteoarthritis or documented previous infective or rheumatic hip conditions. Osteoarthritis groups were scored according to the Harris Hip Score (HHS), Western Ontario and McMaster Universities Arthritis Index (WOMAC), visual analogue scale (VAS) and the radiologic Kellgren–Lawrence (K-L) grading scale. Age, sex and body mass index (BMI) were documented for all patients.

After histological analysis, we divided the OA group of subjects into two subgroups, based on the histological synovitis score according to Krenn [33]—one group consisted of patients with a synovitis score from 0 to 2, and the other group consisted of patients with a synovitis score ≥ 3.

### 4.2. Tissue Collection and Basic Staining Procedures

All surgical procedures (total or partial hip replacement, either for the treatment of OA or femoral neck fracture) were performed with spinal anaesthesia through a posterolateral hip approach with posterior capsule incision. After the extraction of the head and part of the neck of the femur, synovial tissue from the inferior part of the femoral neck adjacent to the femoral head was sampled. Samples were stored in formalin solution containers and sent to the Department of Anatomy, Histology and Embryology of the University of Split School of Medicine for histological analysis. Tissues were formalin-fixed, paraffin-embedded and cut into 5 µm thick sections. Each 10th section was stained with haematoxylin and eosin. The synovial membrane was graded according to Krenn, evaluating three features of chronic synovitis (the enlargement of the lining cell layer, the cellular density of synovial stroma and leukocytic infiltrate) [33]. The detailed characteristics of each group are described in Table 1.

### 4.3. Immunofluorescence Staining

For immunofluorescence, sections were processed through deparaffinisation in xylene, rehydration in decreased ethanol grades and distilled water, as we have described previously [63]. Briefly, sections were heated in a citrate buffer (pH 6.0) for 30 min. After cooling off at room temperature, the slides were rinsed with Phosphate-Buffered Saline (PBS) and coated in blocking buffer (ab64226, Abcam, Cambridge, UK) for a period of 30 min. The following step was overnight incubation at room temperature in a humid chamber with the primary antibodies (Table 2). The slides were washed in PBS the next day, and appropriate secondary antibodies (Table 2) were applied for 1 h. After washing in PBS, the nuclei were stained with DAPI (4′,6-diamidino-2-phenylindole), and samples were mounted in Immu-mount and coverslipped. The slides were examined under a fluorescence microscope (Olympus BX61, Tokyo, Japan) and photographed using a DP71 digital camera (Nikon, Tokyo, Japan) with NIS-Elements F software (version 1).

### 4.4. Data Acquisition and Quantitative Analysis

In order to analyse the expressions of SDC1, SDC2, SDC4, EXT1, EXT2, NDST1 and NDST2, ten non-overlapping fields per sample were taken using 40× objective magnification. To determine the immunofluorescence signal of the observed proteins, we calculated the area percentage that the signal took up in the images, as described previously [63]. Briefly, through using the Lasso tool in Adobe Photoshop (Adobe, San Jose, CA, USA), in all images, the surface layer of the cells (intima) was separated from the underlying tissue (subintima). ImageJ software, version 1.54 (NIH, Bethesda, MD, USA) was used to process images to isolate the positive signal. The resulting images were thresholded using the “triangle” method. The area percentage of the thresholded images was determined using the “analyse particles” function. Significant parts of all analysed images were devoid of any tissue, and a correction of the area percentage was necessary to calculate the actual area percentage. To correct the area percentage value, we determined the number of total pixels (px) of the images and the number of empty space pixels using the Magic Wand tool in Adobe Photoshop. The corrected area percentage that was used for the statistical analyses was calculated using the following formula [64]:

$$Corrected\;area\;percentage=\frac{Uncorrected\;area\;percentage\times total\;px}{total\;px-empty\;space\;px}$$

The statistical analyses were performed using GraphPad Prism version 9.0.0. software (GraphPad Software, San Diego, CA, USA). The results are presented as the mean and standard deviation of the calculated percentages. A two-way analysis of variance (ANOVA) with Tukey’s post hoc test was used to determine the statistical significance of the differences in protein expression between the analysed sample groups. Statistical significance was set at *p* < 0.05.

## 5. Conclusions

In considering the significant socio-economic and medical importance of OA, numerous ways to improve pharmacological interventions are being investigated with the goals of reducing disease progression and pain in patients with OA, including immunomodulators targeting synovitis and pain [65]. Modulating communication between cells in the joint to decrease inflammation represents an attractive approach regarding these goals [66]. In our study, we demonstrated that SDC1 and SDC4, as well as molecules that are involved in syndecan regulation (exostosins, sulfotransferases), showed increased expression in OA-related synovitis in comparison with those in the controls. The interpretation of the results is difficult due to delicate interplay between the investigated molecules and because of their potential pro- and anti-inflammatory effects. Still, we concluded that syndecans (SDC1, SDC4), exostosins (EXT2) and sulfotransferases (NDST1) play a role in the progression and control of synovitis and, consequently, are potential therapeutic targets. Syndecans have already been investigated as a potential therapeutic target for a number of chronic inflammatory diseases [56,67], including OA, but without conclusive results. Regardless, we are of the opinion that our study proves the need for the continuation of such research.

Our study demonstrates that SD1 and SDC4, as well as molecules involved in syndecan regulation (exostosins, sulfotransferases), show increased expression in OA-related synovitis compared with those in controls. Therefore, our data provide more information in support of syndecan (SDC1, SDC4), exostosin (EXT2) and sulfotransferase (NDST1) involvement in the progression and control of synovitis. However, their delicate interplay makes it difficult to conclude their potential pro- and/or anti-inflammatory effects. Further studies are needed to shed more light on their use as potential therapeutic targets.

## Figures and Tables

**Table 1:** Clinical, radiological and pathohistological characteristics of the examined groups.

	Controls	OA Krenn Synovitis Score 0–2	OA Krenn Synovitis Score ≥ 3	* *p* Value
Age (median ± IQR, years)	74 (73.55–76.05)	73 (63.7–75.9)	73 (66–78)	0.854
Sex (male/female)	(6/4)	(7/5)	(6/6)	0.732
BMI (median ± IQR, kg/m^2^)	25.87 (23.97–26.6)	24.7 (23.25–25.82)	26.7 (25.5–29.43)	0.054
K-L grade (median ± IQR)	0.5 (0–1)	2 (2–2)	4 (3–4)	<0.0001
Krenn score (median ± IQR)	0 (0–0)	6.4 (5.6–9)	9 (7–9)	<0.0001
HHS (median ± IQR)	-	48.7 (43.58–56.8)	41 (33.48–49.6)	0.272
VAS (median ± IQR)	-	6 (4.6–6.8)	6 (5–7)	0.784
WOMAC (median ± IQR)	-	46.2 (40.2–56.4)	47.3 (36.1–55.3)	0.918

IQR (interquartile range), OA (osteoarthritis), BMI (body mass index), K-L grade (Kellgren–Lawrence grading scale), HHS (Harris Hip Score), VAS (visual analogue scale), WOMAC (The Western Ontario and McMaster Universities Osteoarthritis Index); * *p* < 0.05, Kruskal–Wallace test.

**Table 2:** Primary and secondary antibodies used in the study.

	Antibodies	Host	Code No.	Dilution	Source
Primary	anti-Sdc1 [B-A38]	Mouse	ab34164	1:100	Abcam, Cambridge, UK
	anti-Sdc2	Rabbit	ab191062	1:200	Abcam, Cambridge, UK
	anti-Sdc4	Rabbit	ab24511	1:100	Abcam, Cambridge, UK
	anti-EXT1	Rabbit	ab126305	1:100	Abcam, Cambridge, UK
	Anti-EXT2	Rabbit	ab102843	1:50	Abcam, Cambridge, UK
	Anti-NDST1	Rabbit	ab151141	1:50	Abcam, Cambridge, UK
	Anti-NDST2	Rabbit	ab1511141	1:100	Abcam, Cambridge, UK
Secondary	Rhodamine Red™-X Anti-Mouse IgG	Donkey	715-295-151	1:400	Jackson Immuno Research Laboratories, Inc., Baltimore, PA, USA
	Alexa Fluor^®^488 Anti-Rabbit lgG	Donkey	711-545-152	1:400	Jackson Immuno Research Laboratories, Inc., Baltimore, PA, USA

## Data Availability

Data will be available upon request.
